# Prophylactic fluoroquinolones in hematopoietic stem cell transplant recipients: A meta-analytic comparison of ciprofloxacin and levofloxacin

**DOI:** 10.1097/MD.0000000000042317

**Published:** 2025-05-09

**Authors:** Mohammed A. Hameed Albalawi, Khaled Mohammed Al-Sayaghi, Samya Mohamed Hegazy, Magda Mubarak Merghani, Nawal M. Osman, Lina O. Abdelmagid, Hanan Mohammed Mohammed, Montaha Mohamed Ibrahim, Ragaa Gasim Ahmed Mohmmed, Zeinab Taha Omer, Hammad Ali Fadlalmola, Sabah Zein Elgendi

**Affiliations:** a Department of Internal Medicine, College of Medicine, Taibah University, AL-Madinah AL-Munawarah, Saudi Arabia; b Department of Medical Surgical Nursing, College of Nursing, Taibah University, Al-Madinah Al-Munawwarah, Saudi Arabia; c Nursing Division, Faculty of Medicine and Health Sciences, Sana’a University, Sana’a, Yemen; d Nursing Department, College of Applied Medical Sciences, Al Qurayat, Jouf University, Saudi Arabia; e Mental Health and Psychiatric Nursing, Collage of Nursing, University of Hafr Albatin, Saudi Arabia; f Department of Family & Community Medicine, Faculty of Medicine, University of Al-Baha, Saudi Arabia; g Clinical Pharmacy, University of Khartoum, Sudan; h Department of Medical-Surgical Nursing, Faculty of Nursing, Al-Baha University, Al-Baha, Saudi Arabia; i Nursing College, King Khalid University, Muhayle Aseer, Saudi Arabia; j Department of Maternal and Child Health Nursing, Faculty of Nursing, Al-Baha University, KSA; k Department of Community Health Nursing, Nursing College, Taibah University, Saudi Arabia; l College of Applied Medical Science, Buraydah Colleges, AL-Qassim, Saudi Arabia; m Faculty of Nursing, Kafrelsheikh University, Egypt.

**Keywords:** ciprofloxacin, hematopoietic stem cell transplant, levofloxacin, meta-analysis, prophylaxis

## Abstract

**Background:**

Hematopoietic stem cell transplantation (HSCT) is a critical therapeutic intervention for hematological malignancies. However, infections remain a leading complication. Fluoroquinolones, particularly ciprofloxacin and levofloxacin, are commonly employed as prophylactic agents. This study compares their efficacy in preventing post-HSCT infections.

**Methods:**

A systematic review and meta-analysis were conducted using the Cochrane and Preferred Reporting Items for Systematic Reviews and Meta-analyses guidelines. Relevant studies comparing ciprofloxacin and levofloxacin in HSCT recipients were identified from biomedical databases. Randomized controlled trials and retrospective cohort studies were included. Data extraction encompassed patient demographics, intervention details, and infection outcomes. Meta-analyses employed RevMan software, using risk ratios (RR) and mean differences with 95% confidence intervals (CI).

**Results:**

Five studies involving 1202 HSCT recipients (597 ciprofloxacin; 605 levofloxacin) were analyzed. Levofloxacin showed superior efficacy in reducing bloodstream infections (BSI) (RR = 1.61; 95% CI: [1.04, 2.49]; *P* = .03) and specifically gram-positive BSI (RR = 1.60; 95% CI: [1.09, 2.36]; *P* = .02). Both agents demonstrated similar effectiveness in preventing febrile neutropenia (RR = 0.99; *P* = .96), gram-negative BSI (RR = 0.99; *P* = .99), pneumonia (RR = 1.24; *P* = .7), and all-cause mortality (RR = 1.05; *P* = .7). Hospital stay duration was also comparable (mean differences = 0.57 days; *P* = .4).

**Conclusions:**

Levofloxacin is more effective in preventing gram-positive BSIs post-HSCT, while ciprofloxacin offers comparable outcomes in other infection-related parameters. Given its broader bacterial coverage and convenient dosing, levofloxacin may be preferred for prophylaxis. Further large-scale randomized trials are recommended to confirm these findings.

## 1. Introduction

Hematopoietic stem cell transplantation (HSCT) is a critical therapeutic intervention for hematological malignancies.^[1]^ Despite its effectiveness, remains a primary source of morbidity and mortality following HSCT.^[2]^ Bloodstream infections (BSI) are particularly common, occurring in 20% to 61% of recipients, often before neutrophil engraftment.^[3,4]^ These infections can lead to prolonged hospital stays and increased healthcare costs.^[5,6]^ Moreover, infection is the leading cause of deaths not related to the primary disease.^[7]^ Several factors contribute to post-HSCT infections, including prolonged neutropenia, mucocutaneous breaks, invasive devices, myeloablative conditioning, prolonged steroid use, and preexisting infections.^[8–11]^

Prophylactic strategies, including antimicrobial administration, have been implemented to reduce infection rates.^[12,13]^ Early studies supported the role of prophylactic antibiotics in reducing febrile episodes post-HSCT.^[14,15]^ A Cochrane meta-analysis revealed significant reductions in all-cause and infection-related mortality with antibiotic use.^[16]^ Current guidelines recommend prophylactic antibiotics from the day of HSCT until neutrophil recovery, especially for patients with expected prolonged neutropenia.^[17–19]^

Fluoroquinolones are characterized by their broad spectrum of bactericidal activity, preservation of the gastrointestinal normal flora, preferable bioavailability, and good tolerance.^[20,21]^ Studies have shown their superiority over placebo and other antibiotics in reducing infection risks post-HSCT.^[22–25]^ Subsequently, numerous guidelines recommended the choice of fluoroquinolones for prophylactic antimicrobial coverage of HSCT recipients.^[19,26–28]^ Specifically, ciprofloxacin and levofloxacin were the most endorsed fluoroquinolones for the prevention of infections after HSCT.^[19]^ Levofloxacin, a newer fluoroquinolone, has broader activity against gram-positive bacteria and anaerobes, making it a preferred choice for prophylaxis.^[29,30]^

This review aims to compare the effectiveness of ciprofloxacin and levofloxacin in preventing post-HSCT infections and their adverse consequences.

## 2. Methods

Throughout the conduct and report of this review, the guidance obtained from Cochranes’ handbook for interventional reviews and the Preferred Reporting Items for Systematic Reviews and Meta-analysis was rigorously followed.^[31,32]^

### 2.1. Literature search and selection of relevant studies

On December 16, 2024, we thoroughly searched biomedical databases from inception, without filters for relevant studies. Our systematic search was carried out using the following terms: ((Ciprofloxacin OR levofloxacin OR fluoroquinolone*) AND (“stem-cell” OR “Stem Cell” OR “Hematopoietic stem cell transplantation” OR HSCT)). All search results were collected and screened after removing the duplicates to select the studies eligible for inclusion in this review. Initially, the authors screened the titles and abstracts of all search results. Thereafter, a comprehensive review of the initially selected studies’ full texts was performed for the final decision of eligibility. The screening was double-checked to ensure accuracy, and the authors discussed any disagreement. Finally, we screened the references lists of all eligible studies in search of further relevant publications.

This review included primary studies that compared the early prophylactic use of ciprofloxacin in comparison with levofloxacin for HSCT recipients. We did not apply restrictions on the source of stem cells, route of administration and dose of ciprofloxacin and levofloxacin, nor on the primary diagnosis or demographics of HSCT recipients. However, animal studies, books, theses, editorials, letters, conference abstracts, and non-English/Arabic studies were not suitable for inclusion in the present review.

### 2.2. Quality assessment

The risk of bias in the conduct and report of randomized controlled trials (RCTs) was evaluated using the tool provided in the Cochranes’ Handbook. Cochrane tool evaluated the probability of bias in selecting the patients for each intervention group, in the provision of the intervention and detection of the outcomes, in patients’ attrition, and in the trial report.^[31]^ On the other hand, cohort studies’ quality was evaluated using the tool designed by the National Institution of Health. Their tool evaluates items regarding the methods used through the conduct of the studies as well as items regarding the studies’ report. According to these items’ judgment, each study is given a total score and a corresponding description of the quality (poor, fair, or good quality).^[33]^

### 2.3. Data extraction

We extracted data on the design, site, sample size, and patients’ inclusion and exclusion criteria of each included study. In addition, we extracted data on the amount of infused CD34 and nucleated cells, the timing of antibiotics prophylaxis, the route of administration and dosing of ciprofloxacin and levofloxacin, and the duration of follow-up applied in each study. Data on the baseline characteristics of HSCT recipients treated with ciprofloxacin were extracted and compared with those of recipients treated with levofloxacin. These baseline data described the recipients’ age, gender, and primary diagnosis, the source of stem cells, the type of conditioning regimen, the administration of granulocyte-colony stimulating factor, and the duration of neutropenia.

The present review primarily explored the effectiveness of ciprofloxacin in comparison with levofloxacin in preventing post-HSCT infection and its adverse consequences. The investigated outcomes included the incidences of febrile neutropenia, BSI, gram-positive BSI, gram-negative BSI, *Clostridium difficile* infection, *Staphylococcus aureus* infection, *Staphylococcus epidermidis* infection, *Streptococcus mitis* infection, vancomycin-resistant *Enterococcus* infection, *Escherichia coli* infection, pneumonia, and all-cause mortality. In addition, we compared the lengths of hospital stay.

### 2.4. Quantitative synthesis

We conducted all our comparative meta-analyses via RevMan software. Continuous and dichotomous outcomes were pooled in the forms of mean difference (MD) and risk ratio (RR) respectively, with the 95% confidence interval (CI) of each pooled estimate. The statistical significance of the pooled estimates was determined when the *P*-value was under .05. All analyses were performed using the fixed-effect model except when a profound level of heterogeneity was noticed (a chi-squared *P*-value under .1 and an I-squared (I^2^) value of 50% or more). In such a case, a random-effect model of analysis was applied.^[34,35]^

### 2.5. Ethical approval

As this study is a systematic review and meta-analysis that does not involve primary data collection or human subjects, ethical approval was not required.

## 3. Results

### 3.1. Literature search and selection of relevant studies

**Figure 1.**
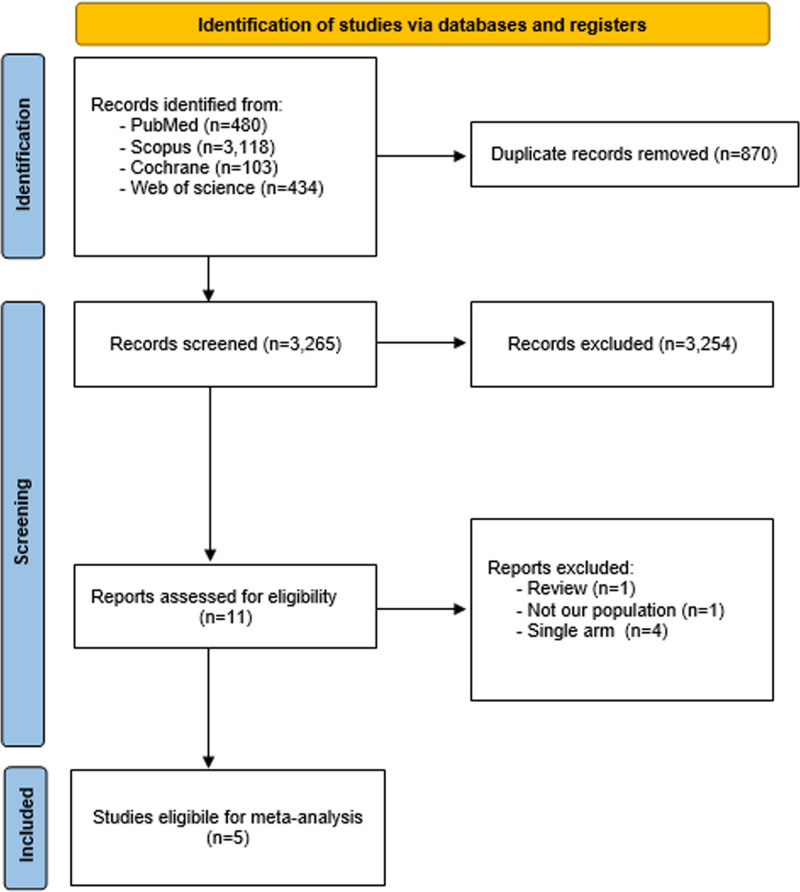
PRISMA flow diagram. PRISMA = Preferred Reporting Items for Systematic Reviews and Meta-analyses.

Our systematic literature search retrieved 4135 results, the majority of which were retrieved from Scopus. After the removal of duplicate results, 3265 publications remained for titles and abstract screening. Of those, eleven studies were initially judged eligible for inclusion. A thorough full-text review of these studies eventually determined the eligibility of 5 studies in the qualitative and quantitative conduct of this review^[36–40]^ (Fig. 1).

### 3.2. Description of the included studies and the enrolled HSCT recipients

The present review included 1 RCT^[36]^ and 4 retrospective cohort studies.^[37–40]^ Three studies were conducted in the United States,^[36,39,40]^ 1 study in Italy,^[37]^ and 1 study in Uruguay.^[38]^ All studies administered the prophylactic fluoroquinolones until neutrophil engraftment was established, starting from the day of HSCT or 1 to 2 days before. Ciprofloxacin was administered orally at a dose of 500 mg twice daily, whereas levofloxacin was administered orally or intravenously at a dose of 500 or 750 mg once or twice daily. Recipients were followed up for 1 to 29 months after HSCT (Table 1).

**Table 1:** Summary of the included studies.

Study ID	Study design	Country	Sample size (ciprofloxacin/levofloxacin)	Eligibility criteria	TNC and CD34 counts infused (cell/Kg)	Ciprofloxacin route and dose	Levofloxacin route and dose	Duration of prophylaxis	Duration of follow-up (months)
Farhan et al (2024)^[36]^	Randomized clinical trial	The United States	308 (154/154)	Patients older than 18 years who received HSCT for hematological malignancies were included. Patients with prolonged QT interval or allergies to ciprofloxacin or levofloxacin were excluded.	TNC: 4.7 × 10^8^ CD34: 4.3 × 10^6^	500 mg, twice daily, orally.	500 mg, once daily, orally.	Started 2 days before HSCT until neutrophil engraftment.	29 ± 10.3
Servidio et al (2021)^[37]^	Retrospective cohort	Italy	180 (60/120)	Patients ≤ 18 years of age who underwent the first attempt of allogeneic HSCT for hematological malignancies and were on a myeloablative conditioning regimen were included. Patients who developed a bacterial infection and received antibiotics near the conditioning regimen and patients who arrived at HSCT with aplasia, severe neutropenia, or fluoroquinolone-resistant germ colonization were excluded.	TNC: 6.1 × 10^8^ CD34: 8.4 × 10^6^	10 mg/kg (maximum 500 mg), twice daily, intravenously.	10 mg/kg (maximum 500 mg), twice daily, intravenously.	Started one day before HSCT until neutrophil engraftment.	3.3
Oliver et al (2020)^[38]^	Retrospective cohort	Uruguay	266 (121/145)	Patients who underwent autologous HSCT for hematological malignancies.	CD34: 4.8 × 10^6^	500 mg, twice daily, orally.	500 mg, once daily.	Started from the day of HSCT until neutrophil engraftment.	3.3
Rambaran et al (2019)^[39]^	Retrospective cohort	The United States	151 (108/43)	Patients aged 18 to 89 years who have cancer and underwent HSCT were included. Patients with a history of fluoroquinolones-resistant infection or fluoroquinolones allergy and incarcerated individuals were excluded.	–	500 mg, twice daily, orally.	750 mg, once daily, orally.	–	–
Copeland et al (2017)^[40]^	Retrospective cohort	The United States	297 (154/143)	Patients who received autologous HSCT for treatment of multiple myeloma.	–	500 mg, twice daily, orally.	500 mg, once daily, orally.	Started from the day of HSCT until neutrophil engraftment.	1

HSCT *=* hematopoietic stem cell transplantation, TNC *=* total nucleated cells.

All the included studies enrolled HSCT recipients with malignancies, hematological malignancies specifically in 4 studies.^[36–38,40]^ A total of 1202 recipients were enrolled in the included studies, 597 received prophylactic ciprofloxacin and 605 received levofloxacin. Four studies enrolled adult recipients who had an average age of 54 to 60 years,^[36,38–40]^ whereas Servidio et al enrolled pediatric recipients with an average age of 8 years.^[37]^ The recipients enrolled in all the included studies were mostly males. The most frequently reported primary diagnoses of the enrolled HSCT recipients were acute lymphoblastic or myeloid leukemia, non-Hodgkin lymphoma, and multiple myeloma (Tables 1 and 2).

**Table 2:** Baseline characteristics of the enrolled recipients.

Study ID	Age (years)		Males		Primary diagnosis												Autologous stem cells source		Chemotherapy-based conditioning		Prophylactic G-CSF		Duration of neutropenia (days)	
					Acute myeloid leukemia		Acute lymphoblastic leukemia		Hodgkin lymphoma		Non-Hodgkin lymphoma		Multiple myeloma		Myelodysplasia									
	Ciprofloxacin	Levofloxacin	Ciprofloxacin	Levofloxacin	Ciprofloxacin	Levofloxacin	Ciprofloxacin	Levofloxacin	Ciprofloxacin	Levofloxacin	Ciprofloxacin	Levofloxacin	Ciprofloxacin	Levofloxacin	Ciprofloxacin	Levofloxacin	Ciprofloxacin	Levofloxacin	Ciprofloxacin	Levofloxacin	Ciprofloxacin	Levofloxacin	Ciprofloxacin	Levofloxacin
	mean ± SD	mean ± SD	n (%)	n (%)	n (%)	n (%)	n (%)	n (%)	n (%)	n (%)	n (%)	n (%)	n (%)	n (%)	n (%)	n (%)	n (%)	n (%)	n (%)	n (%)	n (%)	n (%)	mean ± SD	mean ± SD
Farhan et al (2024)^[36]^	61.7 ± 11.2	62 ± 9.7	88 (57.1%)	84 (54.5%)	–	–	–	–	–	–	–	–	–	–	–	–	95 (61.7%)	102 (66.2%)	-	-	154 (100%)	154 (100%)	11*	11*
Servidio et al (2021)^[37]^	8.5 ± 6.8	8.3 ± 6.8	39 (65%)	76 (64.3%)	57 (47.5%)	14 (11.7%)	30 (50%)	57 (47.5%)	–	–	–	–	–	–	1 (1.7%)	5 (4.2%)	0 (0%)	0 (0%)	36 (60%)	67 (55.8%)	16 (26.7%)	28 (23.3%)	17.7 ± 3.8	16.2 ± 5
Oliver et al (2020)^[38]^	54 ± 8.8	58 ± 8.2	64 (53%)	90 (62%)	7 (5.8%)		6 (4.1%)		19 (15.7%)	15 (10.3%)	35 (29%)	51 (35%)	69 (49.6%)	66 (45.5%)	0 (0%)	0 (0%)	121 (100%)	145 (100%)	121 (100%)	145 (100%)	121 (100%)	145 (100%)	8 ± 2.2	10 ± 3.8
Rambaran et al (2019)^[39]^	58 ± 14.6	56 ± 16.1	71 (65.7%)	22 (51.2%)	7 (6.5%)	4 (9.3%)	9 (8.3%)	4 (9.3%)	6 (5.6%)	4 (9.3%)	22 (20.4%)	12 (27.9%)	56 (51.9%)	17 (39.5%)	4 (3.7%)	1 (2.3%)	77 (71.3%)	31 (72.1%)	108 (100%)	43 (100%)	–	–	6 ± 2.2	6 ± 1.5
Copeland et al (2017)^[40]^	58.7 ± 8.2	60.1 ± 9	85 (55.2%)	79 (55.2%)	0 (0%)	0 (0%)	0 (0%)	0 (0%)	0 (0%)	0 (0%)	0 (0%)	0 (0%)	154 (100%)	143 (100%)	0 (0%)	0 (0%)	154 (100%)	143 (100%)	154 (100%)	143 (100%)	–	–	11.7 ± 0.7	12 ± 1.5

G-CSF = granulocyte-colony stimulating factor, n = number of recipients, SD = standard deviation.

* Medin.

All the patients enrolled by Oliver et al and Copeland et al^[38,40]^ and most of those enrolled by Farhan et al and Rambaran et al^[36,39]^ received autologous HSCT. In contrast, all participants enrolled by Servidio et al received allogeneic HSCT.^[37]^ The count of infused nucleated cells ranged from 4.7 to 6.1 × 10^8^ cells/kg, and that of CD34 cells ranged from 4.3 to 8.4 × 10^6^ cells/kg. All the patients enrolled by Oliver et al, Rambaran et al, and Copeland et al,^[38–40]^ and most of those enrolled by Servidio et al^[37]^ were administered a chemotherapy-based conditioning regimen. Farhan et al reported chemotherapy-based conditioning in some of the recipients as well, but the type of conditioning regimen for the rest of the recipients was not specified.^[36]^ Supportive G-CST was administered for all HSCT recipients enrolled by Farhan et al and Oliver et al^[36,38]^ and some of those enrolled by Servidio et al.^[37]^ Eventually, neutropenia lasted for an average of 6 to 18 days after HSCT (Tables 1 and 2).

### 3.3. Quality evaluation

Farhan et al, the only included RCT, had an overall good quality. Their study used a computerized randomization approach to allocate the recipients to receiving ciprofloxacin or levofloxacin. Farhan et al had an open-label protocol that might have affected the provision of supportive care and treatment of infections but not the provision of intervention medications (preprepared pills). In addition, their open-label protocol is not thought to influence the outcomes detection (objective outcomes). An intention-to-treat analysis was conducted by Farhan et al, minimizing the risk of attrition bias. Moreover, their study had a registered protocol in which, all the pre-specified outcomes were reported in the final published paper.^[36]^

Oliver et al and Rambaran et al^[38,39]^ had a good quality rating, while Servidio et al and Copeland et al^[37,40]^ were judged to have a fair quality. All the included cohort studies were however limited by not including a justification of the sample size nor the power of the study and only assessing the applied intervention once over time. Oliver et al, Servidio et al, and Copeland et al were limited by including the patients in each intervention group from different time intervals.^[37,38,40]^ In addition, Servidio et al and Copeland et al did not account for potential confounders in their analysis.^[37,40]^ In regards to all other evaluated aspects of cohort studies’ conduct, the included studies had good quality (Table S1, Supplemental Digital Content, https://links.lww.com/MD/O858).

### 3.4. Efficacy outcomes

#### 3.4.1. Febrile neutropenia

**Figure 2.**
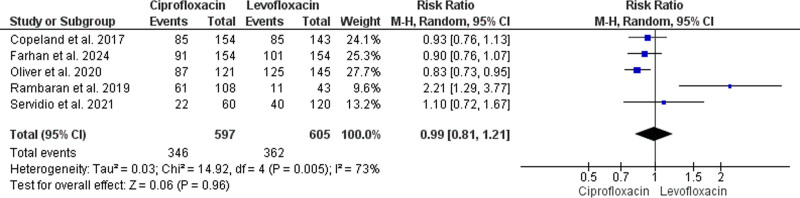
Forest plot of the analysis; febrile neutropenia.

The primary analysis of this outcome was based upon the findings of 5 studies that enrolled 1202 HSCT recipients (597 received ciprofloxacin and 605 received levofloxacin).^[36–40]^ The pooled estimate indicated an insignificant difference between the 2 medications’ effectiveness in preventing febrile neutropenia (RR = 0.99, 95% CI: [0.81, 1.21], *P* = .96). The results pooled in this analysis incorporated a significant level of heterogeneity (*P* = .005, I^2^ = 73%) (Fig. 2).

A sensitivity analysis was subsequently performed by excluding Rambaran et al^[39]^ to resolve the heterogeneity. The findings of this analysis homogenously revealed ciprofloxacin’s superiority over levofloxacin in reducing the risk of febrile neutropenia (RR = 0.88, 95% CI: [0.81, 0.96], *P* = .006) (*P* = .5, I^2^ = 0%) (Figure S1, Supplemental Digital Content, https://links.lww.com/MD/O859).

#### 3.4.2. BSI

**Figure 3.**
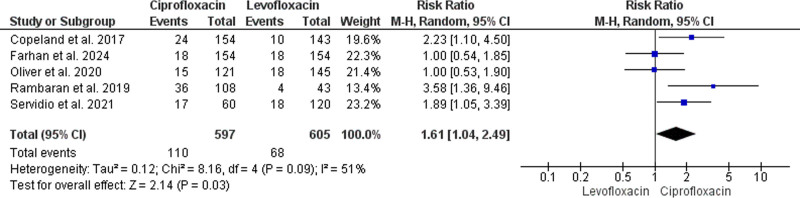
Forest plot of the analysis; bloodstream infections.

The initial analysis of this outcome pooled the results of 5 studies that enrolled a total of 1202 HSCT recipients (597 recipients received ciprofloxacin, while 605 received levofloxacin).^[36–40]^ The pooled estimate indicated more favorable prevention of BSI with levofloxacin administration (RR = 1.61, 95% CI: [1.04, 2.49], *P* = .03); however, variability across the studies’ findings was noticed (*P* = .09, I^2^ = 51%) (Fig. 3).

The results of Rambaran et al^[39]^ were excluded in a subsequent sensitivity analysis which minimized the heterogeneity (*P* = .18, I^2^ = 39%), but yielded an insignificant difference between ciprofloxacin’s and levofloxacin’s effectiveness in preventing BSI (RR = 1.42, 95% CI: [0.95, 2.13], *P* = .09) (Figure S2, Supplemental Digital Content, https://links.lww.com/MD/O859).

#### 3.4.3. Gram-positive BSI

**Figure 4.**
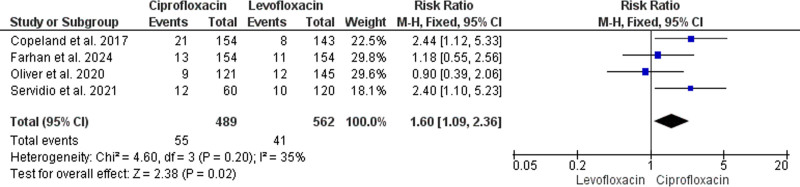
Forest plot of the analysis; gram-positive bloodstream infections.

This comparative meta-analysis included 4 primary studies with 1051 recipients enrolled (489 received prophylactic ciprofloxacin and 562 received levofloxacin).^[36–38,40]^ The analysis homogenously and significantly indicated improved prevention of gram-positive BSI with the prophylactic administration of levofloxacin rather than ciprofloxacin (RR = 1.60, 95% CI: [1.09, 2.36], *P* = .02) (*P* = .2, I^2^ = 35%) (Fig. 4).

#### 3.4.4. Gram-negative BSI

**Figure 5.**
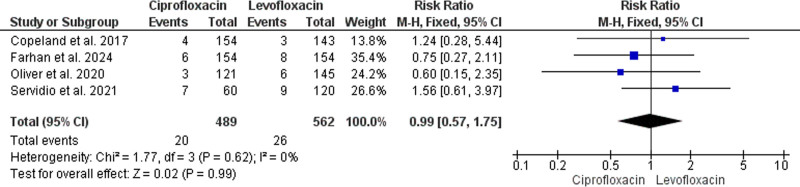
Forest plot of the analysis; gram-negative bloodstream infections.

Both ciprofloxacin and levofloxacin homogenously had similar effectiveness in preventing Gram-negative BSI among HSCT recipients according to our comparative analysis (RR = 0.99, 95% CI: [0.57, 1.75], *P* = .99) (*P* = .6, I^2^ = 0%). This analysis was conducted upon data reported in 4 studies that enrolled 1051 HSCT recipients (489 received ciprofloxacin, whereas 562 received levofloxacin).^[36–38,40]^ (Fig. 5).

#### 3.4.5. Pneumonia

According to our analysis, the effectiveness of the prophylactic administration of ciprofloxacin was similar to that of levofloxacin in regard to preventing pneumonia (RR = 1.24, 95% CI: [0.41, 3.70], *P* = .7), but a profound variability across the studies’ findings was noticed (*P* = .04, I^2^ = 69%). Three studies that enrolled 871 HSCT recipients (429 received ciprofloxacin and 442 received levofloxacin) contributed data to this meta-analysis^[36,38,40]^ (Figure S3, Supplemental Digital Content, https://links.lww.com/MD/O859).

#### 3.4.6. Specific bacterial species infection

We conducted a comparative analysis for the risk of infection with each bacterial species separately. Our meta-analyses concluded a comparable effectiveness of ciprofloxacin and levofloxacin in preventing infections with *C difficile* (RR = 1.52, *P* = .4), *S aureus* (RR = 1.24, *P* = .7), *S epidermidis* (RR = 1.04, *P* = .97), *S mitis* (RR = 63, *P* = .5), vancomycin-resistant *Enterococcus* (RR = 1.21, *P* = .7), and *E coli* (RR = 1.15, *P* = .8). The results of the studies included in all analyses were homogeneous except for the analyses of *C difficile* and *S epidermidis* infections (Table 3, Figures S4–S9, Supplemental Digital Content, https://links.lww.com/MD/O859).

**Table 3:** Prevention of specific bacterial species analysis results.

Bacteria	No. of studies	Ciprofloxacin group (n/N)	Levofloxacin group (n/N)	RR; 95% CI	*P*-value	Heterogeneity
*Clostridium difficile*	5	50/597	32/588	1.52; [0.59, 3.88]	.38	*P* = .007, I^2^ = 72%
*Staphylococcus aureus*	4	7/537	4/485	1.24; [0.40, 3.88]	.71	*P* = .71, I^2^ = 0%
*Staphylococcus epidermidis*	3	22/383	9/342	1.04; [0.10, 11.40]	.97	*P* = .03, I^2^ = 73%
*Streptococcus mitis*	3	2/383	3/342	0.63; [0.15, 2.57]	.52	*P* = .39, I^2^ = 0%
Vancomycin-resistant *Enterococcus*	3	10/416	6/340	1.21; [0.42, 3.46]	.72	*P* = .85, I^2^ = 0%
*Escherichia coli*	4	13/537	10/485	1.15; [0.50, 2.64]	.75	*P* = .63, I^2^ = 0%

CI *=* confidence interval, I^2^ = I-squared, n = event, N = total, RR *=* risk ratio.

The incidence of *Pseudomonas aeruginosa* infection was reported by Farhan et al (in 2 patients who received ciprofloxacin) and Oliver et al (in 1 patient who received levofloxacin).^[36,38]^ Copeland et al reported an insignificant difference between the 2 medications in regard to the incidence of all *Pseudomonas species* infections (*P* = .99).^[40]^ *Klebsiella pneumonia* infection was reported by Farhan et al in 3 patients who received ciprofloxacin.^[36]^ Oliver et al reported *K pneumonia* infection in 2 patients who received ciprofloxacin and 1 patient who received levofloxacin.^[38]^

#### 3.4.7. The duration of hospital stay (days)

**Figure 6.**
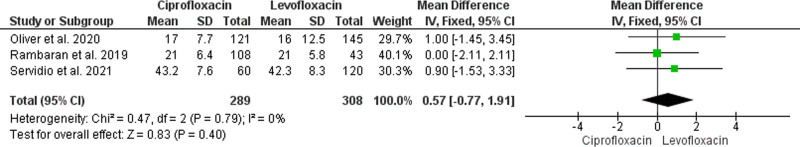
Forest plot of the analysis; the duration of hospital stay (days).

Three studies that reported homogeneous findings of 597 enrolled HSCT recipients (289 recipients received ciprofloxacin, whereas 308 received levofloxacin) were involved in this analysis.^[37–39]^ Transplant recipients who were administered prophylactic ciprofloxacin required a duration of hospitalization that is comparable to those who were administered levofloxacin (MD = 0.57, 95% CI: [-0.77, 1.91], *P* = .4) (*P* = .79, I^2^ = 0%) (Fig. 6).

#### 3.4.8. All-cause mortality

**Figure 7.**
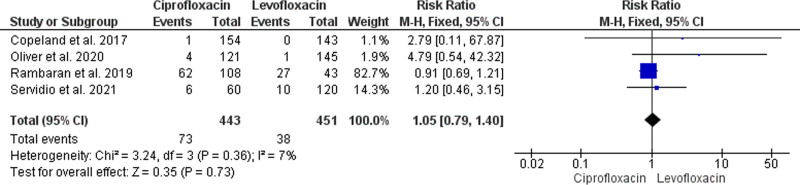
Forest plot of the analysis; all-cause mortality.

Transplant recipients who were prophylactically administered ciprofloxacin had a similar risk of mortality when compared to those who were administered levofloxacin (RR = 1.05, 95% CI: [0.79, 1.40], *P* = .7). This finding is based on our analysis of all-cause mortality risk that pooled the homogeneous findings (*P* = .36, I^2^ = 7%) of 4 studies that collectively enrolled 894 HSCT recipients (443 received ciprofloxacin and 451 received levofloxacin)^[37–40]^ (Fig. 7).

### 3.5. Tolerance

Farhan et al and Servidio et al monitored the recipients for any adverse events related to fluoroquinolone use, specifically, musculoskeletal and cardiac effects. The 2 studies reported the absence of adverse events with ciprofloxacin and levofloxacin use.^[36,37]^

## 4. Discussion

Following HSCT for various indications, infection remains the primary complication responsible for significant morbidity and threat to patients’ lives.^[2]^ Hence, the prophylactic administration of antibiotics, preferably fluoroquinolones, was recommended by the current guidelines from the time of HSCT until neutropenia is resolved.^[19,26–28]^ A survey of 41 transplant centers has reported the prophylactic use of fluoroquinolones in 75% of the centers, where 80% of these centers used levofloxacin and 20% used ciprofloxacin.^[41]^ Studies have compared the prophylactic administration of ciprofloxacin to the newer fluoroquinolone (levofloxacin), yet no consensus has been reached. To this date, a review article has not explored the comparison of the effectiveness of ciprofloxacin versus levofloxacin in preventing post-HSCT infections. Therefore, we constructed this systematic review to summarize the findings of the literature regarding this comparison. In total, data from 1202 HSCT recipients (597 received prophylactic ciprofloxacin and 605 received levofloxacin) were pooled in this review to draw conclusions.

Ciprofloxacin and levofloxacin have structural differences that distinguish each medication’s pharmacokinetics and potency. In comparison with ciprofloxacin, levofloxacin has a more favorable bioavailability.^[30]^ The 2 medications have comparable bactericidal activity against gram-negative bacteria; however, levofloxacin has a broader coverage of gram-positive species and anaerobic bacteria.^[29,30]^ Our analysis revealed the superiority of levofloxacin in reducing the risk of BSI (RR = 1.61, *P* = .03). This superiority was specifically noticed in the prevention of gram-positive BSI (RR = 1.60, *P* = .03), whereas the 2 medications equally prevented gram-negative BSI (*P* = .99). These findings are consistent with the description of levofloxacin’s spectrum of bactericidal activity. In the context of antimicrobial prophylaxis after HSCT, levofloxacin’s prevention of gram-positive infection is of great value. Studies have shown that the majority of post-HSCT infections are attributed to gram-positive bacteria among recipients with hematological malignancies as well as those with solid tumors. Specifically, coagulase-negative *Staphylococci* and alpha-hemolytic *Streptococci* are the most frequently identified organisms.^[42,43]^ Nevertheless, our analysis did not indicate a significant difference between ciprofloxacin and levofloxacin in preventing *S epidermidis* or *S mitis* infections. In addition, the risks of acquiring *S aureus*, *C difficile*, and vancomycin-resistant *Enterococcus* infections were comparable with the 2 medications. The superior effectiveness of levofloxacin in preventing gram-positive infections might manifest in preventing other species that are not investigated in this review. Our findings in regard to the similar risk of acquiring gram-negative infection with *E coli* is consistent with the description of ciprofloxacin’s and levofloxacin’s comparable bactericidal coverage of gram-negative bacteria. Due to the scarcity of data, we could not perform separate analyses for the risks of *P aeruginosa* and *K pneumonia* infections which are leading causes of severe sepsis and death.^[44,45]^

Recent trends in antimicrobial stewardship have raised questions about the routine use of fluoroquinolone prophylaxis in HSCT recipients. Trecarichi et al (2023) reported that omitting fluoroquinolone prophylaxis was associated with a significant increase in gram-negative BSI, particularly with colonizing strains.^[46]^ Akhmedov et al (2023) reported that omitting fluoroquinolone prophylaxis was associated with a significant increase in gram-negative BSI, particularly with colonizing strains.^[47]^ Similarly, Akhmedov and Espinoza (2024) highlighted that while antimicrobial stewardship programs are essential to combat resistance, the abrupt discontinuation of fluoroquinolone prophylaxis may lead to increased infection-related morbidity, especially in high-risk HSCT recipients.^[48]^ These findings underscore the importance of judicious use of prophylactic fluoroquinolones while considering patient-specific risk factors. Our current analysis supports the continued value of fluoroquinolone prophylaxis, particularly levofloxacin, in preventing BSI in HSCT recipients. Future studies should focus on risk-stratified approaches to antimicrobial prophylaxis that balance infection prevention with antimicrobial stewardship concerns.

Levofloxacin is described as having extensive absorption and distribution through body systems, including deep respiratory tissues.^[49]^ Yet, our analysis did not detect a significant difference between levofloxacin and ciprofloxacin in preventing pneumonia (*P* = .7). Moreover, the incidence of febrile neutropenia was similar among HSCT recipients who were administered ciprofloxacin and those who were administered levofloxacin (*P* = .96). The similar risk of febrile neutropenia despite BSI prevention with levofloxacin suggests the presence of other factors that contribute to fever. According to our analysis, pneumonia is not 1 of these factors as its risk was comparable with the 2 medications administration. Besides several previous studies,^[50–53]^ both Farhan et al and Oliver et al^[36,38]^ have reported the contribution of fever of unknown origin to the incidence of febrile neutropenia. Among Oliver et al’s HSCT recipients, fever without a known origin was the most frequently reported complication after HSCT.^[38]^ Furthermore, our review did not analyze the differences in the risks of urinary tract infections, central-line-related infections, and other potential causes of fever due to the unavailability of data in the included studies. In accordance with our finding regarding febrile neutropenia, the comparative analyses revealed an insignificant difference in the duration of hospitalization (*P* = .4) and the risk of mortality (*P* = .7) between patients who received levofloxacin and those who received ciprofloxacin.

Although levofloxacin was only found superior to ciprofloxacin in reducing BSI (gram-positive BSI specifically), this feature is clinically valuable. Reducing BSI relieves the morbidity burden on the immuno-compromised HSCT recipients and minimizes the utilization of diagnostic and therapeutic healthcare resources.^[51]^ A cost-analysis study estimated that the healthcare of each BSI costs over 40,000$.^[54]^ Thus, choosing levofloxacin over ciprofloxacin for prophylaxis after HSCT comes with significant economic benefits. One of the major concerns with the prophylactic use of fluoroquinolones is the emergence of fluoroquinolone-resistant bacterial strains.^[55,56]^ However, fluoroquinolone resistance is reversible.^[57,58]^ Moreover, based on our findings, we suggest that BSI prevention with levofloxacin would reduce the need for frequent empirical antibiotics administration to treat BSI. This would subsequently reduce the possibility of resistance to the empirical antibiotics, which is more significant clinically. Furthermore, unlike ciprofloxacin, levofloxacin has favorable bioavailability and penetration and a longer duration of action, which allows for convenient, once daily, dosing.^[29,30]^

The present review is distinguished by being the first to explore the comparison between ciprofloxacin and levofloxacin for prophylaxis after HSCT. The primary studies included in this review were RCT and cohort studies which provide the most reliable evidence of interventional and observational studies respectively. Moreover, these studies had an acceptable quality. The performed quantitative synthesis further adds to the credibility of the evidence provided in the present review. However, a considerable level of heterogeneity was noticed across the results pooled in some of the analyses. Primarily, Rambaran et al’s findings were different from those of other studies.^[39]^ This is thought to originate from the primary diagnosis of patients enrolled in their study. In contrast to other studies that enrolled recipients with hematological malignancies specifically, Rambaran et al enrolled HSCT recipients with various forms of cancer (including hematological ones). Unlike other studies as well, Rambaran et al administered levofloxacin at a dose of 750 mg (vs 500 mg in other studies). When the study of Rambaran et al was excluded in sensitivity analyses of the risks of febrile neutropenia and BSI, the significance of the pooled estimate changed. The sensitivity analyses indicated a superior effectiveness of ciprofloxacin in preventing febrile neutropenia and a similar effectiveness of the 2 medications in regard to BSI prevention. On the other hand, Servidio et al enrolled pediatric patients who received allogeneic HSCT, administered levofloxacin twice daily intravenously, and administered ciprofloxacin in combination with cotrimoxazole. Despite these variations, Servidio et al’s results were generally consistent with that of the other included studies.^[37]^ In all cases, we accounted for heterogeneity in the pooled estimate calculation by applying a random-effect model analysis. In addition, a sensitivity analysis was performed whenever applicable. The present review is further limited by the small number of patients enrolled. Also, due to the scarcity of available data, we could not analyze the differences between ciprofloxacin and levofloxacin in regard to the site of infection (e.g., the central venous line, urinary tract, and the colon), certain causes of infection (such as *P aeruginosa* and *K pneumonia*), the need for a higher level of care, and the risk of infection-related mortality. We recommend conducting further research, preferably RCTs on a large scale, to address the limitations and support the evidence presented in the present review. Future studies are recommended to stratify their results by the primary diagnosis, the source of stem cells, the type of conditioning regimen applied, and other factors that might influence the outcomes in order to draw case-specific conclusions.

## 5. Conclusion

This review concludes the superior efficacy of the prophylactic administration of levofloxacin when compared to ciprofloxacin in preventing BSI, specifically gram-positive BSI, after HSCT. Otherwise, the 2 medications had a similar efficacy in preventing febrile neutropenia, pneumonia, and all-cause mortality as well as a similar duration of hospital stay. Further research on a larger scale is required to support and add to the evidence presented in this review.

## Acknowledgments

The authors would like to thank the Deanship of Research and Graduate Studies at King Khalid University for their funding support through the Large Research Project (grant number RGP2/366/45).

## Author contributions

**Conceptualization:** Mohammed ِ A. Hameed Albalawi.

**Data curation:** Khaled Mohammed Al-Sayaghi, Samya Mohamed Hegazy, Nawal M. Osman, Lina O. Abdelmagid, Montaha Mohamed Ibrahim, Ragaa Gasim Ahmed Mohmmed, Zeinab Taha Omer, Sabah Zein Elgendi.

**Formal analysis:** Hammad Ali Fadlalmola, Khaled Mohammed Al-Sayaghi, Nawal M. Osman, Hanan Mohammed Mohammed, Montaha Mohamed Ibrahim, Ragaa Gasim Ahmed Mohmmed, Sabah Zein Elgendi.

**Methodology:** Magda Mubarak Merghani, Ragaa Gasim Ahmed Mohmmed, Zeinab Taha Omer.

**Resources:** Magda Mubarak Merghani, Nawal M. Osman.

**Software:** Samya Mohamed Hegazy, Lina O. Abdelmagid, Sabah Zein Elgendi.

**Supervision:** Hammad Ali Fadlalmola.

**Writing – original draft:** Mohammed ِ A. Hameed Albalawi, Hammad Ali Fadlalmola, Samya Mohamed Hegazy, Hanan Mohammed Mohammed, Montaha Mohamed Ibrahim, Zeinab Taha Omer.

**Writing – review & editing:** Mohammed ِ A. Hameed Albalawi, Hammad Ali Fadlalmola, Khaled Mohammed Al-Sayaghi, Magda Mubarak Merghani, Lina O. Abdelmagid, Hanan Mohammed Mohammed, Montaha Mohamed Ibrahim, Ragaa Gasim Ahmed Mohmmed, Sabah Zein Elgendi.

## Supplementary Material


