# Supplementary material for: Prophylactic fluoroquinolones in hematopoietic stem cell transplant recipients: A meta-analytic comparison of ciprofloxacin and levofloxacin
Source: Medicine (Baltimore). 2025 May 9;104(19):e42317. doi: 10.1097/MD.0000000000042317 (PMC12074066; doi:10.1097/MD.0000000000042317)
Supplement: Supplementary file 1 [file medi-104-e42317-s001.pdf]

Table S.1: Quality assessment of cohort studies.

Study ID	Was the research question or objective in this paper clearly stated?	Was the study population clearly specified and defined?	Was the participation rate of eligible persons at least 50%?	Were all the subjects selected or recruited from the same or similar populations (including the same time period)? Were inclusion and exclusion criteria for being in the study prespecified and applied uniformly to all participants?	Was a sample size justification, power description, or variance and effect estimates provided?	For the analyses in this paper, were the exposure(s) of interest measured prior to the outcome(s) being measured?	Was the time frame sufficient so that one could reasonably expect to see an association between exposure and outcome if it existed?	For exposures that can vary in amount or level, did the study examine different levels of the exposure as related to the outcome (e.g. categories of exposure, or exposure measured as continuous variable)?	Were the exposure measures (independent variables) clearly defined, valid, reliable, and implemented consistently across all study participants?	Was the exposure(s) assessed more than once over time?	Were the outcome measures prespecified, clearly defined, valid, reliable, and assessed consistently across all study participants?	Were the people assessing the outcomes blinded to the participants' exposures/interventions?	Was the loss to follow-up after baseline 20% or less? Were those lost to follow-up accounted for in the analysis?	Were key potential confounding variables measured and adjusted statistically for their impact on the relationship between exposure(s) and outcome(s)?	Quality rating
**Servidio et al. 2021 [1]**	Yes	Yes	Yes	No	No	Yes	Yes	NA	Yes	No	Yes	No	Yes	No	**Fair**
**Oliver et al. 2020 [2]**	Yes	Yes	Yes	No	No	Yes	Yes	NA	Yes	No	Yes	No	Yes	Yes	**Good**
**Rambaran et al. 2019 [3]**	Yes	Yes	Yes	Yes	No	Yes	Yes	NA	Yes	No	Yes	No	Yes	Yes	**Good**
**Copeland et al. 2017 [4]**	Yes	Yes	Yes	No	No	Yes	Yes	NA	Yes	No	Yes	No	Yes	No	**Fair**

Each question is answered: Yes=1, No=0.5, Not Reported (NR), Cannot Determine (CD) or Not Applicable (NA)=0. Quality rating: good (11-14 points) or fair (7.5-10.5 points) or poor (0-7 points).

References

1. Servidio AG, Simeone R, Zanon D, Barbi E, Maximova N. Levofloxacin Versus Ciprofloxacin-Based Prophylaxis during the Pre-Engraftment Phase in Allogeneic Hematopoietic Stem Cell Transplant Pediatric Recipients: A Single-Center Retrospective Matched Analysis. Antibiotics (Basel, Switzerland). 2021;10(12). 10.3390/antibiotics10121523
2. Oliver AC, Riva E, Mosquera R, Galeano S, Pierri S, Bello L, et al. Comparison of two different anti-infectious approaches after high-dose chemotherapy and autologous stem cell transplantation for hematologic malignancies in a 12-year period in British Hospital, Uruguay. Annals of hematology. 2020;99(4):877-84. 10.1007/s00277-020-03947-1
3. Rambaran KA, Seifert CF. Ciprofloxacin vs. levofloxacin for prophylaxis in recipients of hematopoietic stem cell transplantation. Journal of oncology pharmacy practice : official publication of the International Society of Oncology Pharmacy Practitioners. 2019;25(4):884-90. 10.1177/1078155218787286
4. Copeland V, McLaughlin M, Trifilio S. Ciprofloxacin vs levofloxacin for prophylaxis during hematopoietic stem-cell transplantation. Clinical transplantation. 2018;32(1). 10.1111/ctr.13145
