# Supplementary material for: Prophylactic fluoroquinolones in hematopoietic stem cell transplant recipients: A meta-analytic comparison of ciprofloxacin and levofloxacin
Source: Medicine (Baltimore). 2025 May 9;104(19):e42317. doi: 10.1097/MD.0000000000042317 (PMC12074066; doi:10.1097/MD.0000000000042317)

Figure S.1: Forest plot of the analysis; Febrile neutropenia (sensitivity analysis).

Study or Subgroup	Ciprofloxacin Events	Ciprofloxacin Total	Levofloxacin Events	Levofloxacin Total	Weight	Risk Ratio M-H, Random, 95% CI
Copeland et al. 2017	85	154	85	143	20.8%	0.93 [0.76, 1.13]
Farhan et al. 2024	91	154	101	154	26.4%	0.90 [0.76, 1.07]
Oliver et al. 2020	87	121	125	145	48.2%	0.83 [0.73, 0.95]
Rambaran et al. 2019	61	108	11	43	0.0%	2.21 [1.29, 3.77]
Servidio et al. 2021	22	60	40	120	4.6%	1.10 [0.72, 1.67]
**Total (95% CI)**		**489**		**562**	**100.0%**	**0.88 [0.81, 0.96]**
Total events	285		351			

Heterogeneity: Tau² = 0.00; Chi² = 2.35, df = 3 (P = 0.50); I² = 0%
Test for overall effect: Z = 2.76 (P = 0.006)

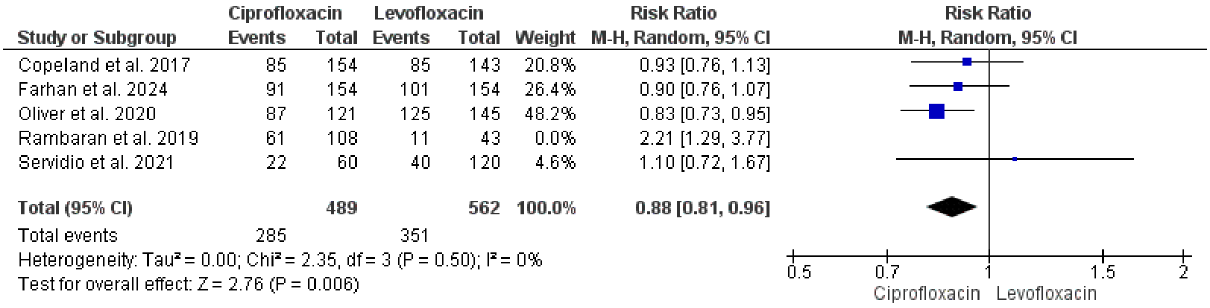

Figure S.2: Forest plot of the analysis; Bloodstream infections (sensitivity analysis).

Study or Subgroup	Ciprofloxacin Events	Ciprofloxacin Total	Levofloxacin Events	Levofloxacin Total	Weight	Risk Ratio IV, Random, 95% CI
Copeland et al. 2017	24	154	10	143	22.0%	2.23 [1.10, 4.50]
Farhan et al. 2024	18	154	18	154	26.0%	1.00 [0.54, 1.85]
Oliver et al. 2020	15	121	18	145	24.6%	1.00 [0.53, 1.90]
Rambaran et al. 2019	36	108	4	43	0.0%	3.58 [1.36, 9.46]
Servidio et al. 2021	17	60	18	120	27.4%	1.89 [1.05, 3.39]
**Total (95% CI)**		**489**		**562**	**100.0%**	**1.42 [0.95, 2.13]**
Total events	74		64			

Heterogeneity: Tau² = 0.07; Chi² = 4.91, df = 3 (P = 0.18); I² = 39%
Test for overall effect: Z = 1.69 (P = 0.09)

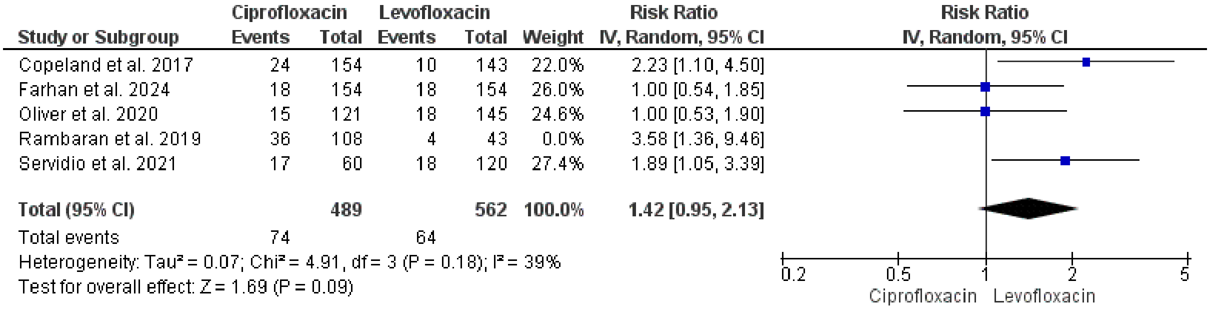

Figure S.3: Forest plot of the analysis; Pneumonia.

Study or Subgroup	Ciprofloxacin Events	Ciprofloxacin Total	Levofloxacin Events	Levofloxacin Total	Weight	Risk Ratio M-H, Random, 95% CI
Copeland et al. 2017	3	154	2	143	21.7%	1.39 [0.24, 8.22]
Farhan et al. 2024	18	154	7	154	38.7%	2.57 [1.11, 5.98]
Oliver et al. 2020	8	121	17	145	39.6%	0.56 [0.25, 1.26]
**Total (95% CI)**		**429**		**442**	**100.0%**	**1.24 [0.41, 3.70]**
Total events	29		26			

Heterogeneity: Tau² = 0.62; Chi² = 6.54, df = 2 (P = 0.04); I² = 69%
Test for overall effect: Z = 0.38 (P = 0.71)

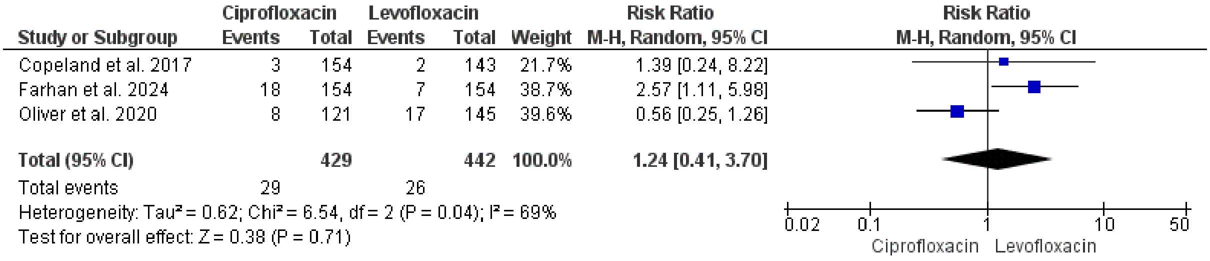

Figure S.4: Forest plot of the analysis; *Clostridium difficile* infection.

	Ciprofloxacin		Levofloxacin			Risk Ratio
Study or Subgroup	Events	Total	Events	Total	Weight	M-H, Random, 95% CI
Copeland et al. 2017	8	154	14	126	23.4%	0.47 [0.20, 1.08]
Farhan et al. 2024	12	154	8	154	23.1%	1.50 [0.63, 3.57]
Oliver et al. 2020	3	121	5	145	17.4%	0.72 [0.18, 2.95]
Rambaran et al. 2019	18	108	2	43	17.3%	3.58 [0.87, 14.79]
Servidio et al. 2021	9	60	3	120	18.8%	6.00 [1.69, 21.35]
**Total (95% CI)**		**597**		**588**	**100.0%**	**1.52 [0.59, 3.88]**
Total events	50		32			

Heterogeneity: Tau² = 0.80; Chi² = 14.15, df = 4 (P = 0.007); I² = 72%
Test for overall effect: Z = 0.87 (P = 0.38)

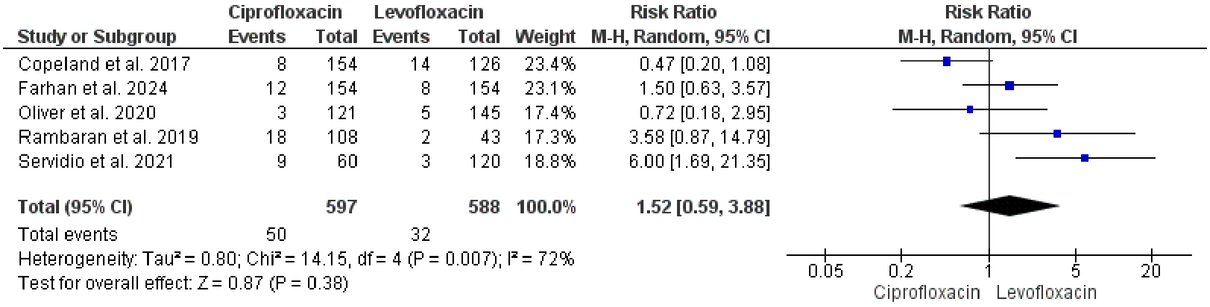

Figure S.5: Forest plot of the analysis; *Staphylococcus aureus* infection.

	Ciprofloxacin		Levofloxacin			Risk Ratio
Study or Subgroup	Events	Total	Events	Total	Weight	M-H, Fixed, 95% CI
Copeland et al. 2017	2	154	2	143	38.6%	0.93 [0.13, 6.51]
Farhan et al. 2024	2	154	0	154	9.3%	5.00 [0.24, 103.30]
Oliver et al. 2020	0	121	1	145	25.4%	0.40 [0.02, 9.70]
Rambaran et al. 2019	3	108	1	43	26.6%	1.19 [0.13, 11.17]
**Total (95% CI)**		**537**		**485**	**100.0%**	**1.24 [0.40, 3.88]**
Total events	7		4			

Heterogeneity: Chi² = 1.39, df = 3 (P = 0.71); I² = 0%
Test for overall effect: Z = 0.38 (P = 0.71)

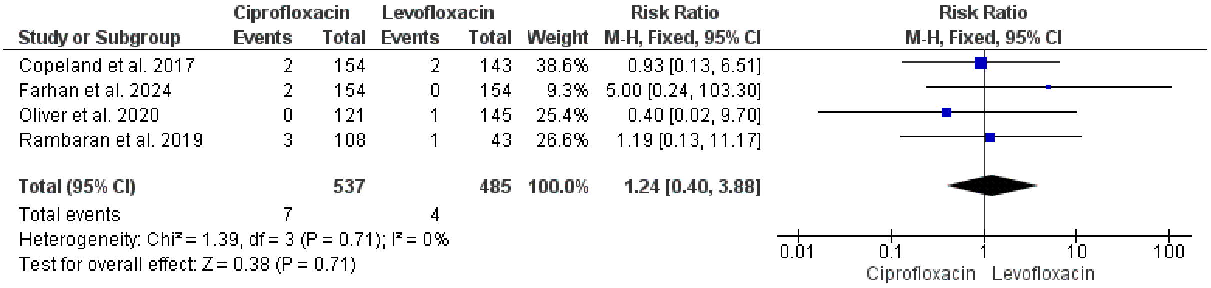

Figure S.6: Forest plot of the analysis; *Staphylococcus epidermidis* infection.

	Ciprofloxacin		Levofloxacin			Risk Ratio
Study or Subgroup	Events	Total	Events	Total	Weight	M-H, Random, 95% CI
Farhan et al. 2024	1	154	4	154	33.4%	0.25 [0.03, 2.21]
Oliver et al. 2020	2	121	5	145	38.1%	0.48 [0.09, 2.43]
Rambaran et al. 2019	19	108	0	43	28.4%	15.74 [0.97, 255.09]
**Total (95% CI)**		**383**		**342**	**100.0%**	**1.04 [0.10, 11.40]**
Total events	22		9			

Heterogeneity: Tau² = 3.22; Chi² = 7.38, df = 2 (P = 0.03); I² = 73%
Test for overall effect: Z = 0.03 (P = 0.97)

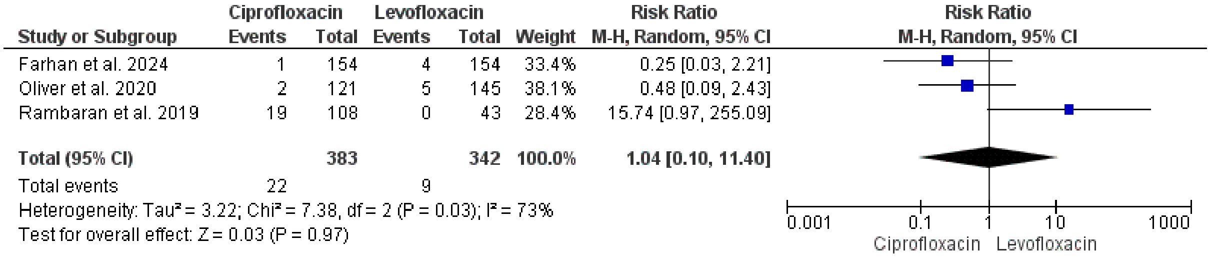

Figure S.7: Forest plot of the analysis; *Streptococcus mitis* infection.

Study or Subgroup	Ciprofloxacin Events	Ciprofloxacin Total	Levofloxacin Events	Levofloxacin Total	Weight	Risk Ratio M-H, Fixed, 95% CI
Farhan et al. 2024	2	154	1	154	22.2%	2.00 [0.18, 21.83]
Oliver et al. 2020	0	121	1	145	30.3%	0.40 [0.02, 9.70]
Rambaran et al. 2019	0	108	1	43	47.5%	0.13 [0.01, 3.24]
**Total (95% CI)**		**383**		**342**	**100.0%**	**0.63 [0.15, 2.57]**
Total events	2		3			
Heterogeneity: Chi² = 1.88, df = 2 (P = 0.39); I² = 0%						
Test for overall effect: Z = 0.65 (P = 0.52)						

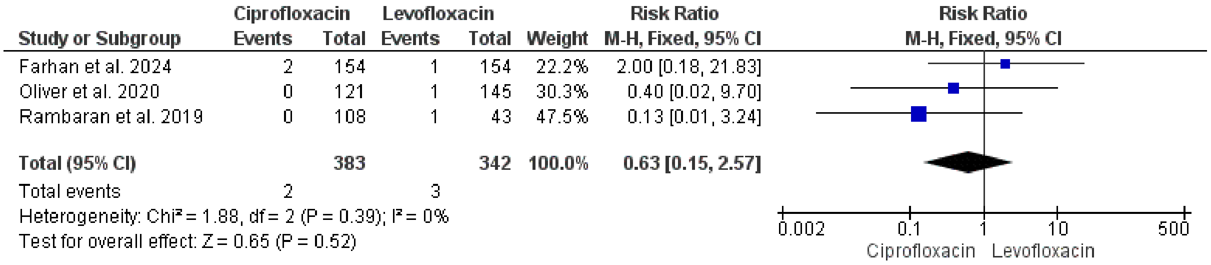

Figure S.8: Forest plot of the analysis; Vancomycin-resistant *Enterococcus* infection.

Study or Subgroup	Ciprofloxacin Events	Ciprofloxacin Total	Levofloxacin Events	Levofloxacin Total	Weight	Risk Ratio M-H, Fixed, 95% CI
Copeland et al. 2017	1	154	1	143	16.0%	0.93 [0.06, 14.71]
Farhan et al. 2024	4	154	4	154	61.8%	1.00 [0.25, 3.93]
Rambaran et al. 2019	5	108	1	43	22.1%	1.99 [0.24, 16.55]
**Total (95% CI)**		**416**		**340**	**100.0%**	**1.21 [0.42, 3.46]**
Total events	10		6			
Heterogeneity: Chi² = 0.32, df = 2 (P = 0.85); I² = 0%						
Test for overall effect: Z = 0.35 (P = 0.72)						

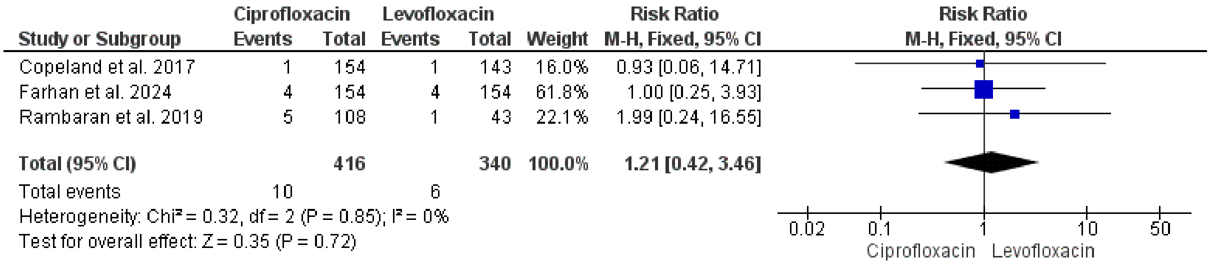

Figure S.9: Forest plot of the analysis; *Escherichia coli* infection.

Study or Subgroup	Ciprofloxacin Events	Ciprofloxacin Total	Levofloxacin Events	Levofloxacin Total	Weight	Risk Ratio M-H, Fixed, 95% CI
Copeland et al. 2017	2	154	2	143	19.9%	0.93 [0.13, 6.51]
Farhan et al. 2024	2	154	4	154	38.4%	0.50 [0.09, 2.69]
Oliver et al. 2020	5	121	4	145	34.9%	1.50 [0.41, 5.45]
Rambaran et al. 2019	4	108	0	43	6.8%	3.63 [0.20, 66.07]
**Total (95% CI)**		**537**		**485**	**100.0%**	**1.15 [0.50, 2.64]**
Total events	13		10			
Heterogeneity: Chi² = 1.75, df = 3 (P = 0.63); I² = 0%						
Test for overall effect: Z = 0.32 (P = 0.75)						

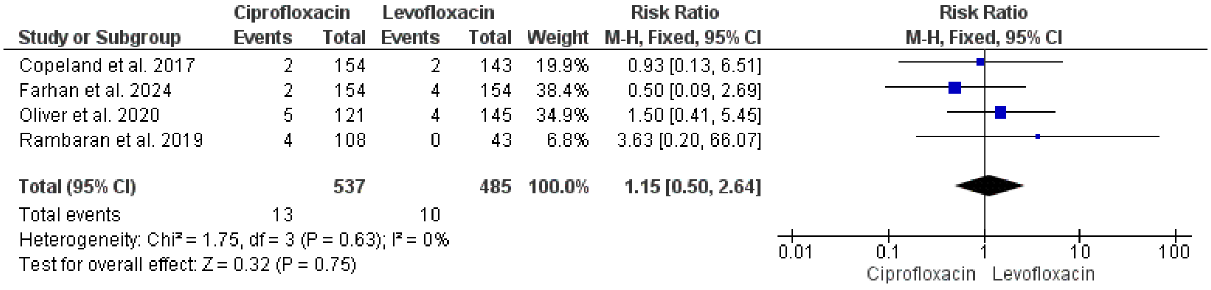

Supplement: Supplementary file 2 [file medi-104-e42317-s002.pdf]
